# Is Morning Exposure Therapy More Successful? Pilot Data on Exposure Process Indicators and Diurnal Cortisol in Obsessive–Compulsive Disorder or Agoraphobia

**DOI:** 10.3390/jcm15176735

**Published:** 2026-08-30

**Authors:** Michael Kellner, Maja Santai Mareljic, Alexander Yassouridis

**Affiliations:** 1 Department of Psychiatry and Psychotherapy, TUM University Hospital Klinikum Rechts Der Isar, 81675 Munich, Germany; 2 Max Planck Institute of Psychiatry, 80336 Munich, Germany

**Keywords:** exposure therapy, cortisol, circadian, obsessive–compulsive disorder, agoraphobia

## Abstract

**Background/Objectives:** Glucocorticoids have been shown to improve fear extinction learning in preclinical and human studies. We hypothesized an impact of the circadian rhythm of cortisol on exposure therapy in patients with anxiety spectrum disorders. **Methods:** Sixteen patients suffering from obsessive–compulsive disorder and eight with agoraphobia participated in a pilot randomized prospective cohort study. During two therapist-guided sessions of exposure therapy with response prevention, both starting either at 08:00 (morning group) or at 16:00 (late afternoon group), subjective units of distress (SUD) and salivary cortisol levels were assessed repeatedly. **Results:** While expected significant diurnal differences in salivary cortisol emerged between daytime groups, after controlling for chronotype, no significant differences were detected in our exposure process indicators of within-session and between-session habituation and distress-related expectancy violation towards maximal SUD and SUD at end. Nearly one-hundred subjects in each group would be needed to gain statistically significant daytime differences for these indicators as per case number estimation. **Conclusions:** Our preliminary results from this transdiagnostic sample do not provide support for scheduling exposure sessions in the morning. Further research should consider cortisol administration before exposure sessions to test whether different doses of exogenous glucocorticoids can augment exposure therapy outcomes.

## 1. Introduction

Exposure therapy is considered as gold standard in the treatment of anxiety spectrum disorders, such as obsessive–compulsive disorder (OCD), agoraphobia, other phobias, post-traumatic stress disorder and generalized anxiety disorder [1,2]. In exposure with response prevention (ERP), patients face previously avoided objects, activities, situations or thoughts without performing behaviors that they used to reduce associated distress. Both habituation of distress and distress-related expectancy violation are discussed as underlying mechanisms [3]. Several strategies to further enhance the efficacy of exposure therapy have been tested and discussed in recent years, among them additional administration of pharmacological agents potentially enhancing fear extinction, e.g., d-cycloserine or pregnenolone [4,5]. One respective agent is the glucocorticoid cortisol, which may augment consolidation of fear extinction memory and inhibit fear memory retrieval [6]. In rodents, a time-of-day difference in fear extinction learning, which is dependent on adrenal endogenous glucocorticoids, was demonstrated [7]. In humans, endogenous cortisol secretion shows a circadian rhythm with a zenith in the morning and a nadir in the evening. Instead of treating patients with pharmacologically administered cortisol, adjusting exposure therapy times according to the diurnal maximum of hypothalamic–pituitary–adrenocortical axis activity could boost fear extinction in patients. Interestingly, previous research has shown that extinction of conditioned fear in healthy human subjects as an experimental analog for exposure therapy is better learned in the morning than in the evening [8]. Translating these results to a clinical sample of patients with spider phobia undergoing one session exposure therapy, significantly better treatment outcomes one week after exposure in the morning than in the evening were observed, which were traced back to higher cortisol levels [9].

Regarding patients suffering from OCD, so far only two studies about cortisol and exposure therapy outcomes have been published. In an exploratory analysis in a small subsample the authors of the present study previously could not find that cortisol during exposure predicts therapy outcomes after one month [10]. Reanalyzing these data, no significant correlation of cortisol levels during exposure and within-session habituation as an early outcome indicator was shown in fifty-one OCD patients [11]. However, these two studies were performed in the afternoon, when cortisol levels already are quite low, because our group primarily tried to replicate our previous finding of non-response of cortisol to exposure therapy [12]. Therefore, another study maximizing variation in endogenous cortisol levels by testing a morning group (with high cortisol levels) versus a late afternoon group was initiated. Focusing on the first two exposure sessions in a mixed population of OCD and agoraphobic patients, the influence of time of day on different exposure process indicators, i.e., intra-session habituation, expectancy violation toward the maximum arousal and expectancy violation toward arousal at the end of the session during the first exposure session, as well as inter-session habituation between sessions one and two was studied. These indicators have been shown to potentially predict therapy outcomes during the further course [3]. However, in the current study, emphasis was placed on habituation and distress-related expectancy violation as target observational variables and clinical outcomes were not measured. Controlling for morningness–eveningness, better short-term exposure session outcomes in the morning versus the later afternoon across anxiety spectrum disorders were hypothesized and the number of subjects needed to observe statistically significant differences in the above-mentioned indicators between groups was estimated.

## 2. Methods and Materials

### 2.1. Subjects

After screening 144 subjects, 24 adult patients suffering from obsessive–compulsive disorder or agoraphobia (according to DSM-5 criteria) were recruited for this interim analysis in our out-patient department for obsessions/compulsions and anxiety. They had to be motivated to complete a short-term 10-session psychotherapeutic intervention including two identical guided ERP sessions (both either in the morning or in the late afternoon). Study inclusion diagnosis and psychiatric comorbidity were assessed by the Standardized Clinical Interview for DSM-5 axis I [13]. Antidepressant drugs were permitted, if they had been on a stable dose for at least three weeks. Exclusion criteria were acute suicidality, a psychotic disorder, organic brain syndrome, substance abuse, gravidity, epilepsy, diabetes mellitus, glucocorticoid therapy, and heart or pulmonary disease.

The study was conducted in accordance with the Declaration of Helsinki, and approved by the Ethics Committee of the university hospital of the Technical University Munich (study number 2022-463-S-KK). It has been recorded in the WHO-acknowledged German Clinical Trials Register (DRKS #00031341). Each subject provided written consent prior to participation after oral and written study information.

### 2.2. Psychotherapeutic and Psychometric Study Procedures

During the first four 60 min sessions, psychoeducation about OCD or agoraphobia, individual behavioral analysis (including a 0–100 OCD or agoraphobia hierarchy indicating putative arousal provoked by items in case of response prevention) and elaboration of the exposure rationale (discussing habituation and expectancy violation as potentially helpful) were performed. These sessions were not standardized by daytime. OCD patients filled in the Dimensional Obsessive–Compulsive Scale [14] and the Yale-Brown Obsessive–Compulsive Scale [15] was applied. Agoraphobic patients filled in the Panic and Agoraphobia Scale [16]. Chronotype was assessed with the Morningness–Eveningness Questionnaire (MEQ) [17] and depression with the Beck Depression Inventory [18] in all patients.

Two identical guided sessions including exposure with response prevention (each of which were 120 min in duration and took place within one week) were conducted either at 07:30 (exposure start 08:00) or at 15:30 (exposure start 16:00) according to 1:1 randomization (in blocks of two for index diagnosis/sex). The exposure item chosen had to be rated at least 70 on the OCD or agoraphobia hierarchy. During the initial 30 min, anticipatory anxiety was assessed, final questions of the patients about the ERP procedure were clarified and the exposure places were approached. During the 90 min of ERP, patients were in constant contact with the avoided stressor, either in vivo or for the exposure item aggressive thoughts in sensu. The attending trained therapists helped to prevent overt and covert avoidance and neutralization behavior.

A 100 mm visual analog scale was utilized for measuring subjective units of distress (SUD), for morning exposure at 07:30, 08:00, 08:05, 08:15, 08:30, 09:00, and 09:30, and for late afternoon exposure at 15:30, 16:00, 16:05, 16:15, 16:30, 17:00, and 17:30. Furthermore, subjects were asked just before exposure to speculate about maximal SUD rating during upcoming exposure and SUD rating before the respective ending.

Three further 60 min sessions were performed to coach the patients to prepare and debrief further unassisted exposure sessions and a tenth session at 3 months for follow-up.

### 2.3. Cortisol Assessment

Salivary samples to assess the free, unbound, biologically active fraction of cortisol were taken at the same times as SUD ratings. All subjects received oral and written instructions on the correct use of the “Salivette” salivary collection device (Sarstedt AG, Nuernbrecht, Germany). Participants were advised not to eat, drink or smoke during exposure sessions. Salivary specimens were centrifuged and supernatants stored until analysis at −85 °C. A commercial enzyme immunoassay was used (IBL International GmbH, Hamburg, Germany). Samples were run in duplicates. Inter- and intra-assay coefficients of variation were below 10%.

### 2.4. Statistics

The effects of “daytime” (morning versus late afternoon) and of “session” (first versus second ERP) on the indicator AUC (area under the curve) of the courses of salivary cortisol and of SUD ratings were evaluated by analyses of variance with repeated measures design. Thereby, “daytime” was considered a between-subjects factor and “session” a within-subjects factor, with MEQ (chronotype) ratings as a covariate.

For investigating habituation and expectancy violation on SUD assessments, we defined the following exposure process indicators: a. “within-session habituation” as maximum SUD rating minus the minimal SUD rating after the start of exposure 1; b. “between-session habituation” as maximum SUD rating after the start of exposure 1 minus the maximum SUD rating after the start of exposure 2; c. “expectancy violation towards maximal SUD” as expected maximal SUD before session 1 minus actual maximal SUD in session 1; and d. “expectancy violation towards SUD at end” as expected SUD at end before session 1 started minus actual SUD at end of session 1. Here, multivariate analyses of variance with “daytime” as between-subjects factor and MEQ (chronotype) as a covariate were performed (in two separate analyses on habituation and expectancy violation parameters, respectively).

Differences between morning versus evening group in demographic, psychometric, clinical and exposure session continuous variables were measured using multiple analysis of variance followed by post hoc F-tests in case of significant global effects. For nominal variables, Fisher’s exact test was applied to test group effects on them.

As nominal level of significance alpha = 0.05 was accepted. Data are given as mean ± SEM.

## 3. Results

### 3.1. Study Sample

The final study sample is shown in Table 1. Statistically significant differences between morning and afternoon exposure group were demonstrated for none of the reported measures. One OCD patient and one agoraphobic patient were comorbid with panic disorder, as was one OCD patient with generalized anxiety disorder. Three patients took escitalopram; each one was on sertraline, fluoxetine, clomipramine, trazodone or hypericin.

Four of the 24 initially recruited patients (three women, one man; three with OCD with predominant contamination fear, one with agoraphobia, i.e., in public transportation) withdrew consent before the first exposure session. Respective reasons were high anticipatory anxiety (in two patients), admission to a hospital close to home and unwillingness to give up morbid gain. One agoraphobic patient only participated in the first exposure session because of hospitalization due to a fractured ankle one day before session two.

### 3.2. Cortisol Levels in Saliva

**Figure 1:**
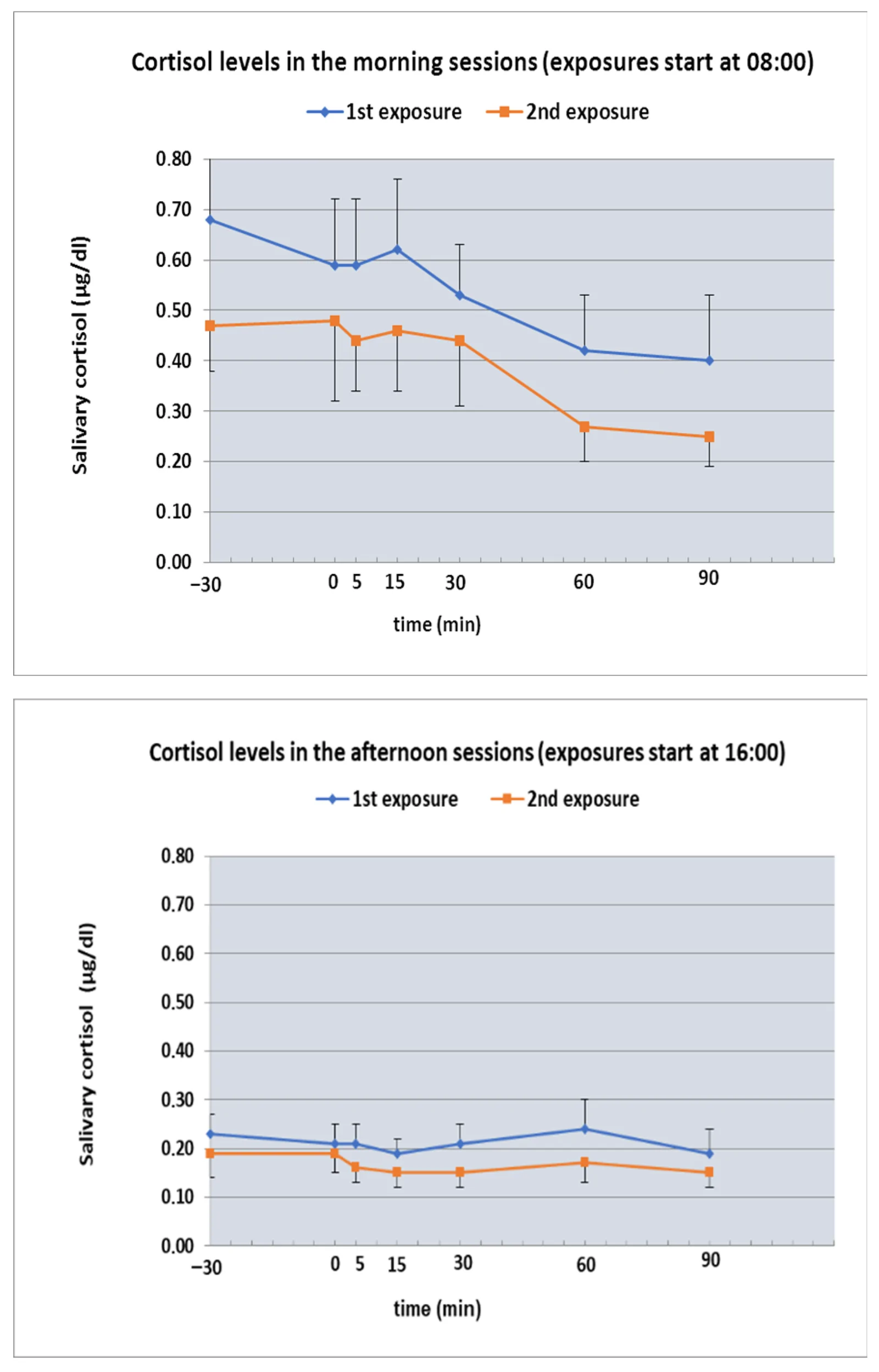
Salivary cortisol (mean ± SEM) during morning and afternoon exposure sessions.

The time–concentration graphs for salivary cortisol are shown in Figure 1.

AUC cortisol during the first exposure session was 12.63 ± 2.66 (n = 9) in the morning group versus 5.15 ± 0.89 (n = 11) in the later afternoon group. During the second exposure session, AUC cortisol was 9.22 ± 2.33 (n = 8) in the morning and 3.76 ± 0.72 (n = 11) in the late afternoon. Analysis of variance revealed a significant daytime effect (F(1,16) = 11.01, *p* = 0.004) with elevated cortisol in the morning and a significant session effect (F(1,17) = 5.75, *p* = 0.028) with higher concentration in the first session, but no significant daytime × session interaction (F(1,17) = 1.28, *p* = 0.273). Also, no effect of the covariate chronotype (MEQ) was found (F(1,16) = 1.61, *p* = 0.223).

### 3.3. Subjective Units of Distress (SUD)

**Figure 2:**
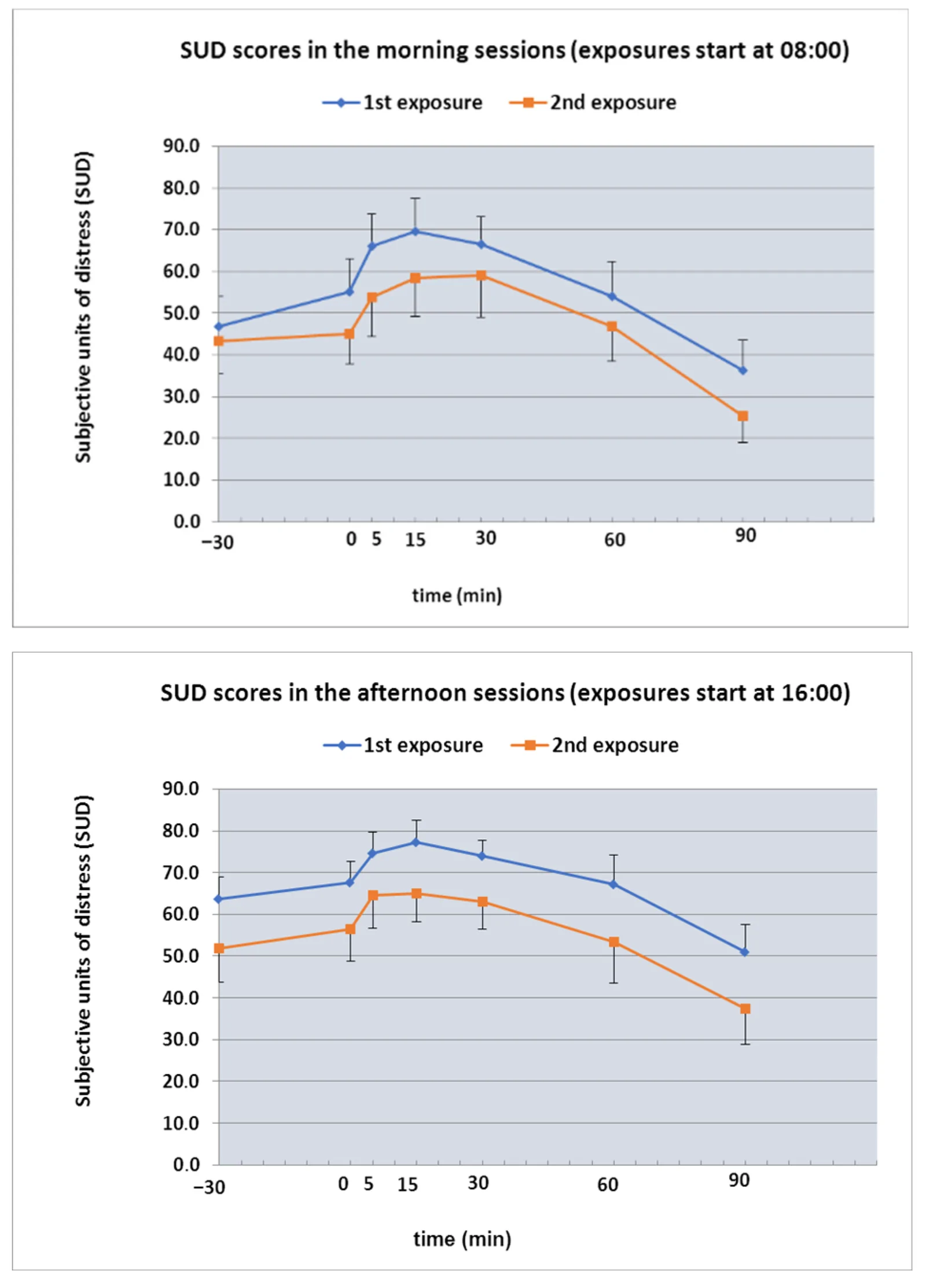
Subjective units of distress (mean ± SEM) during morning and afternoon exposure sessions.

The graphs for SUD during exposures are shown in Figure 2.

AUC SUD in the first exposure was 1338.94 ± 142.84 (n = 9) in the morning and 1620.23 ± 97.02 (n = 11) in the late afternoon. In the second exposure AUC SUD was 1137.25 ± 176.58 (n = 8) in the morning group versus 1579.50 ± 181.00 (n = 11) in the afternoon. As for cortisol, a significant session effect on the AUC of the SUD ratings was detected by analysis of variance (F(1,17) = 6.84, *p* = 0.018); AUC was significantly higher during the first session. But in contrast to cortisol, the daytime effect on the SUD ratings was not significant (F(1,16) = 1.39, *p* = 0.256). No daytime × session effect was found (F(1,17) = 0.60, *p* = 0.449) and no effect of chronotype as a covariate (F(1,16) = 0.01, *p* = 0.924).

### 3.4. Exposure Process Indicators

The results for the four predefined process indicators of the exposure sessions, i.e., within-session habituation during exposure session 1, between-session habituation between sessions 1 and 2, and the expectation violations regarding maximal distress and distress at the end of session 1, are shown in Table 2.

On the two habituation parameters, no significant daytime effect was found by multiple analysis of variance (Wilk’s multivariate test of significance; effect of daytime: F(2,15) = 0.562, *p* = 0.581). Neither within-session habituation (univariate F-test: F(1,16) = 0.070, *p* = 0.795) nor between-session habituation (univariate F-test: F(1,16) = 1.200, *p* = 0.289) showed significant differences. The covariate MEQ also showed no significant influence on the results of the habituation parameters (F(2,15) = 0.692, *p* = 0.516).

Furthermore, for the two distress-related expectancy violation parameters, no significant daytime effect was evident, neither on expectancy violation towards maximal SUD (F(1,17) = 0.678, *p* = 0.422) nor on the expectancy violation towards SUD at end (F(1,17) = 0.493, *p* = 0.492). Again, MEQ as a covariate had no significant influence (F(3,26) = 0.293, *p* = 0.827).

For respective effect sizes and estimations of case numbers needed to detect significant effects between daytime groups (power 80%), please see Table 2.

## 4. Discussion

Despite significant expected differences between morning and late afternoon cortisol levels, no significant effects of daytime on exposure process indicators were detected in our interim analysis. While within-session habituation was nominally higher in the morning, between-session habituation was even greater in the later afternoon in contrast to our hypothesis. However, expectancy violation parameters were nominally higher in the morning, as expected. Regardless, none of these indicators reached statistically significant differences between daytime groups in our small sample. And case number estimation showed that nearly one-hundred subjects per daytime group would be needed to gain statistical significance, which does not provide strong support for scheduling clinical exposure sessions in the morning.

The relationship between endogenous cortisol levels and exposure therapy outcomes has been studied in several anxiety spectrum disorders. Siegmund et al. [19] showed a consistent relationship between lower cortisol concentrations and less therapeutic improvement after three in vivo exposures (flooding) were performed in ten patients with panic disorder and agoraphobia in the early afternoon. Meuret et al. [20] reported in twenty-six patients with panic with agoraphobia that higher cortisol levels during exposure were related to faster rates of clinical improvement in the course of four exposure sessions, which were distributed across the day. Reanalyzing former data demonstrated that early-day extinction-based therapy sessions yield better outcomes than later-day sessions, and were significantly mediated by cortisol levels [21]. However, a meta-analysis on these studies and additional research in specific phobias [22,23] and in an adolescent sample with mixed anxiety disorders [24] did not confirm basal cortisol or cortisol during exposure session to predict significantly better therapy outcome [25]. Conversely, in social anxiety disorder significantly worse outcome of exposure therapy in subjects with higher cortisol levels during sessions was reported [26]. Recently, the group that published the seminal study on the “morning exposure effect” [9] was unable to replicate their previous finding in another study in a specific phobia (now in eighty snake-phobic subjects) and concluded that potential diurnal effects on exposure therapy might be more complex than previously assumed [27].

Psychologically stressful exposure sessions do not cause noteworthy elevations of cortisol during treatment in OCD [10,11], as also seen in the present study’s data. This pattern of cortisol unresponsiveness is also observed during exposure therapy in agoraphobia [19], as well as during lactate-induced panic attacks in panic disorder [28]. A contraintuitive absent or blunted endocrine response is also observed in other psychosocial and biological stress paradigms in panic [29,30]. In exposure, phobic fear cortisol levels remain unaffected [31]. Therefore, prior administration of exogenous cortisol to increase cortisol levels in anxiety spectrum disorders would be an option for potentially beneficial clinical actions on fear extinction learning without the danger of running into ceiling effects. Indeed, double-blind placebo-controlled studies in social phobia and spider phobia have shown that oral administration of cortisol (10 or 25 mg) one hour before exposure resulted in a significantly facilitated extinction of phobic fear [32]. Also, in patients with height phobia, enhancement of extinction-based psychotherapy by administration of 20 mg of cortisol one hour before treatment sessions was demonstrated [33]. However, such studies need to be performed in patients with OCD or agoraphobia and would greatly contribute to testing clinical relevance of enhancing cortisol in exposure-based psychotherapy.

The focus of the present study was on the effects on habituation and distress-related expectancy violation during the first two identical exposure sessions; therefore, we did not standardize treatment thereafter. Continuing self-exposure reliably either in the morning or the afternoon/evening did not seem adequately controllable and differences in motivation and persistence for steady therapeutic exercises would bring additional variance to the data. Of course, early clinical follow-up ratings using a disorder specific questionnaire, such as DOCS, Y-BOCS or PAS, would have yielded valuable additional information. Although a recent study showed in OCD patients that the early outcome parameters for exposure sessions that were used in this current study are a significant predictor for treatment benefits after twenty sessions [3], other investigations in anxiety spectrum disorders showed inconsistent results, especially for habituation parameters [34,35,36,37]. Further shortcomings of our study include the low sample size and the transdiagnostic sample with differences in neurobiology and symptom structure. The small sample does not yet allow meaningful subgroup analyses for diagnosis or gender. Furthermore, we did not assess childhood trauma history, menstrual status, smoking, sleep quality, vigilance, attentional control, cognitive/emotional traits and individual coping skills. Furthermore, our SUD ratings had a low temporal resolution and more frequent sampling points could be considered. Alternative exposure process indicators such as slope-based SUD changes or model-based trajectories across all SUD observations were not regarded. Nevertheless, our subjects represent a real-world sample of motivated patients participating in a clinically pragmatic study with a translational hypothesis.

Future studies about pharmacological effects of different doses of cortisol on exposure therapy outcomes in anxiety spectrum disorders should control for chronotype, as we did, and in addition assess sleep and vigilance [27]. In addition, we advise to further characterize the study subjects before treatment regarding individual glucocorticoid receptor function, e.g., by a dexamethasone suppression test and by measurement of lymphocyte glucocorticoid receptor number and function as a proxy for brain glucocorticoid receptor sensitivity [38,39]. Whether a more sophisticated approach in a considerably larger sample leads to a stratified further optimization of exposure therapy outcomes by additional glucocorticoid treatment needs further investigation.

## Figures and Tables

**Table 1:** Final study sample (mean ± SEM or number).

	Morning (08:00) Exposure (n = 9)	Afternoon (16:00) Exposure (n = 11)
**Demographic & clinical characteristics:**		
Age (years)	33.2 ± 3.7	27.8 ± 2.1
Women/men	5/4	6/5
Obsessive–compulsive disorder	6	7
Agoraphobia	3	4
Comorbid major depression	6	5
No antidepressant drugs	6	6
**Psychometric ratings:**		
Morningness–Eveningness Questionnaire	54.0 ± 2.5	51.0 ± 3.1
Dimensional Obsessive–Compulsive Scale	24.8 ± 5.2	24.7 ± 4.0
Yale-Brown Obsessive–Compulsive Scale	22.3 ± 3.0	21.7 ± 2.5
Panic and Agoraphobia Scale	23.0 ± 2.7	24.3 ± 2.0
Beck Depression Inventory	12.9 ± 2.0	21.3 ± 2.0
**Exposure sessions:**		
Exposure item hierarchy (0–100)	85.0 ± 3.5	87.7 ± 2.4
Exposure item contamination	3	3
Exposure item aggressive thoughts	2	2
Exposure item control behavior	1	2
Exposure item subway ride	3	4
First exposure session completed	9	11
Second exposure session completed	8	11

**Table 2:** Exposure process indicators (mean ± SEM) during morning and afternoon exposure sessions, respective effect sizes and case number estimation.

	Morning (08:00) Exposure (n = 9)	Afternoon (16:00) Exposure (n = 11)	Effect Size (Cohen’s d)	N Needed (per Group)
Within-session habituation (session 1)	39.44 ± 8.96	38.09 ± 4.91	0.062	3218
Between-session habituation (sessions 1–2)	9.50 ± 4.33	17.36 ± 4.89	0.382	86
Expectancy violation towards maximal SUD (session 1)	8.78 ± 6.72	2.91 ± 2.90	0.385	85
Expectancy violation towards SUD at end (session 1)	12.22 ± 11.34	−0.27 ± 10.00	0.375	89

## Data Availability

Dataset available from the authors on request.
